# PEBRA trial – effect of a peer-educator coordinated preference-based ART service delivery model on viral suppression among adolescents and young adults living with HIV: protocol of a cluster-randomized clinical trial in rural Lesotho

**DOI:** 10.1186/s12889-020-08535-6

**Published:** 2020-03-30

**Authors:** Thabo Ishmael Lejone, Mathebe Kopo, Nadine Bachmann, Jennifer Anne Brown, Tracy Renée Glass, Josephine Muhairwe, Tebatso Matsela, Ramona Scherrer, Lebohang Chere, Tilo Namane, Niklaus Daniel Labhardt, Alain Amstutz

**Affiliations:** 1 SolidarMed, Partnerships for Health, Lesotho; 2 Department of Medicine, Clinical Research Unit, Swiss Tropical and Public Health Institute, Socinstrasse 57, 4051 Basel, Switzerland; 3 University of Basel, Basel, Switzerland; 4 Molecular Virology, Department of Biomedicine, University of Basel, Basel, Switzerland; 5 Sentebale, Lesotho; 6 District Health Management Team, Butha Buthe, Lesotho; 7 Motebang Government Hospital, Leribe, Lesotho; 8 Department of Infectious Diseases and Hospital Epidemiology, University Hospital Basel, Basel, Switzerland

**Keywords:** HIV, Adolescent, Lesotho, Africa, southern, Randomized controlled trial, Peer group, Antiretroviral therapy, differentiated service delivery

## Abstract

**Background:**

Despite tremendous progress in controlling the HIV epidemic in sub-Saharan Africa, HIV-related mortality continues to increase among adolescents and young people living with HIV (AYPLHIV). Globally, sub-Saharan Africa accounts for 85% of the AYPLHIV. Overall outcomes along the HIV care cascade are worse among AYPLHIV as compared to all other age groups due to various challenges in accessing and adhering to antiretroviral therapy (ART). New, innovative multicomponent packages of differentiated service delivery (DSD) models, are required to address the specific needs of AYPLHIV. This study aims to evaluate the feasibility and effectiveness of a multicomponent DSD model (PEBRA model) designed for AYPLHIV and coordinated by a peer-educator.

**Methods:**

PEBRA (Peer-Educator Based Refill of ART) is a cluster randomized, open-label, superiority trial conducted at 20 health facilities in three districts of Lesotho, Southern Africa. The clusters (health facilities) are randomly assigned to either the PEBRA model or standard of care in a 1:1 ratio, stratified by district. AYPLHIV aged 15–24 years old in care and on ART at one of the clusters are eligible. In the PEBRA model, a peer-educator coordinates the antiretroviral therapy (ART) services - such as medication pick-up, SMS notifications and support options - according to the preferences of the AYPLHIV. The peer-educator delivers this personalized model using a tablet-based application called PEBRApp. The control clusters continue to offer standard of care: ART services coordinated by the nurse. The primary endpoint is viral suppression at 12 months. Secondary endpoints include self-reported adherence to ART, quality of life, satisfaction with care and engagement in care. The target sample size is 300 AYPLHIV. Statistical analyses are conducted and reported in line with CONSORT guidelines for cluster randomized trials.

**Discussion:**

The PEBRA trial will provide evidence on the feasibility and effectiveness of an inclusive, holistic and preference-based DSD model for AYPLHIV that is coordinated by a peer-educator. Many countries in SSA have an existing peer-educator program. If proven effective, the PEBRA model and PEBRApp have the potential to be scaled up to similar settings.

**Trial registration:**

Clinicaltrials.gov, NCT03969030. Registered on 31 May 2019. More information: www.pebra.info

## Background

There is encouraging progress towards an AIDS-free generation by 2030 on a global scale. However, this progress is challenged by persistent poor outcomes among young people in sub-Saharan Africa (SSA). SSA accounts for 85% of the adolescents and young people living with HIV (AYPLHIV) worldwide. Almost one third of new HIV infections occur among individuals aged 15–25 years, mostly in females [1, 2]. AYPLHIV is the only population group for whom HIV-related mortality continues to increase and they are more likely to drop out of HIV care, and have overall worse health outcomes than all other age groups, especially in rural areas [3–7]. AYPLHIV face particular challenges in accessing and adhering to ART. The distinct rapid physical, psychological and emotional changes that occur during adolescence impact on how AYPLHIV perceive their health, make decisions, handle risks and interact with health and related services [8]. Thus, barriers in the adolescent HIV care cascade are multifactorial [9–13]. Multicomponent packages of differentiated service delivery (DSD) are a promising approach to address these multiple barriers [14, 15]. Unlike service delivery models that apply standardized care for all people living with HIV, the idea of DSD models is to consider the specific needs of a group of people, while facilitating service scale-up by reducing the burden on health systems and increasing efficiency [16, 17]. In 2018, Paediatric-Adolescent Treatment Africa, in collaboration with other key stakeholders, undertook a situational analysis of DSD for AYPLHIV in South Africa [18]. They report a lack of published literature documenting adolescent-specific DSD models in the Southern African region. Moreover, the analysis shows that most adolescents are not accessing DSD models even where they exist, an indicator that the existing DSD models are not tailored according to adolescent-specific preferences.

Many countries in SSA are expanding their peer-educator (PE) program and the World Health Organization (WHO) and International AIDS Society highly recommend engagement of peers in service delivery [19, 20]. The question arises to which extent PEs can be involved in coordinating DSD models and ART services for their peers. Some evidence exists, including a systematic review showing that PE involvement leads to improved engagement in care, psychosocial well-being and HIV knowledge, however, PE involvement is usually limited to support group interventions in urban settings with an unclear effect on biological outcomes [21–23]. One recent cluster-randomized clinical trial from rural Zimbabwe demonstrated a positive impact of peer-led services on psychosocial as well as virological outcomes for AYPLHIV [24, 25].

Lesotho has the second-highest HIV prevalence in the world with and adult prevalence of 25.6% and one of the highest HIV incidences among adolescent girls and young women [1, 26]. According to the recent household-based national survey (LePHIA) overall viral suppression in AYPLHIV is 50.9% among female and 46.1% among male [27]. In close collaboration with different local stakeholders, we designed a DSD model specifically for AYPLHIV, called the PEBRA model, built upon existing support structures in Lesotho and delivered by a dedicated tablet application. In the PEBRA model, PEs first assess the ART service preferences of their peers, and then differentiate the services according to these preferences in a feasible manner.

## Methods/design

### Setting

The PEBRA trial will be conducted in northern Lesotho, in the districts of Leribe, Butha-Buthe and Mokhotlong. All districts are characterized by mostly rural settings, poor transport infrastructure and hard-to-reach villages with a combined estimated population of ca. 550′000. Butha-Buthe district comprises 12 public/missionary health facilities, Leribe district 26, and Mokhotlong district 10 that offer ART services. The HIV prevalence among individuals 15–59 years old ranges from 17.8% in Butha-Buthe, to 23.7% in Leribe and 26.1% in Mokhotlong [27].

### Design

PEBRA trial is a cluster randomized, open-label, superiority trial in rural Lesotho. The clusters (health facilities) are randomized into two groups (intervention and control) in a 1:1 allocation stratified by district. Cluster randomization was applied to eliminate the risk of cross-contamination between the study arms.

### Eligibility and randomization

Eligible clusters are public or missionary nurse-led health facilities (not hospitals) that offer ART services, serve a rural population, are situated in an area with stable cell phone signal and have a PE who passes the study-specific training assessment. Randomization is stratified by district due to differences in viral suppression rates across the districts. To obtain consent and to maximize transparency and ownership of the health facilities, randomization events involving all health facilities and the District Health Management Team were conducted in each district. At these events, health facility representatives drew opaque, sealed, equally sized envelopes containing the group allocation from a Mokorotlo (traditional Lesotho hat) and disclosure took place only once all facilities had drawn their envelope. To further ensure allocation concealment and to minimize potential selection bias, the sequence of drawing was randomly selected in advance by an independent person drawing from a second pile of opaque, sealed envelopes containing the names of the facility.

The PEs actively screen all young people at their facility for inclusion. Eligible individuals are attending care at a participating facility, take ART, 15–24 years old, and provide written informed consent.

### Control clusters

Participants in the control clusters receive the standard of care offered in Lesotho at nurse-led rural health facilities. The ART visits and refills are clinic-based and coordinated by the nurse. Some of the clinics have functioning clinic or community support clubs as well as SMS notifications to inform when VL results are available.

### Intervention clusters

**Fig. 1:**
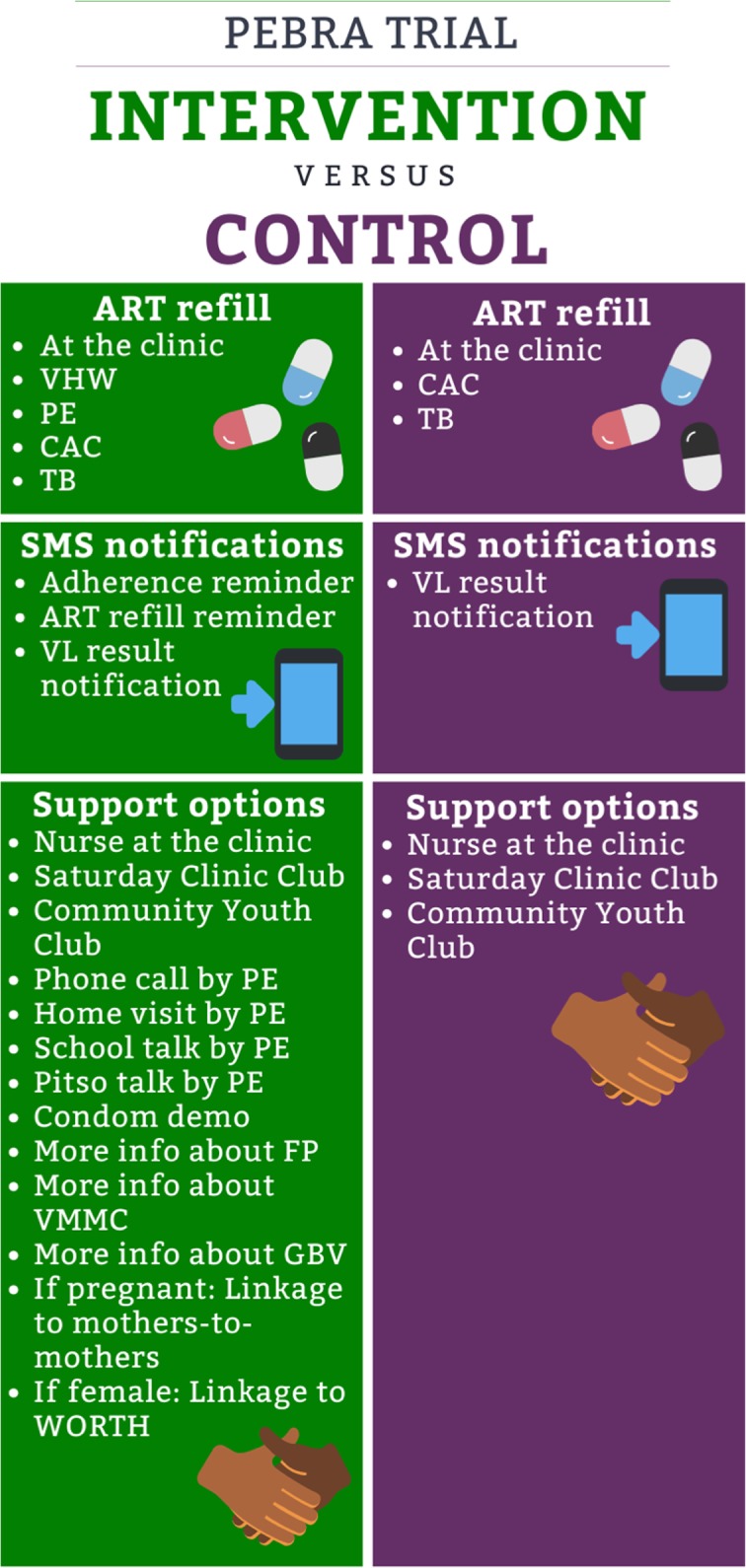
PEBRA intervention versus PEBRA control. ***Abbreviations:*** VHW (Village Health Worker), PE (Peer-Educator), CAC (Community Adherence Club), TB (Treatment Buddy), ART (antiretroviral therapy), VL (viral load), FP (Family Planning), VMMC (Voluntary Medical Male Circumcision), GBV (Gender-Based Violence), WORTH (Sentebale Social Asset Building Model). “Pitso” = Village gathering

**Fig. 2:**
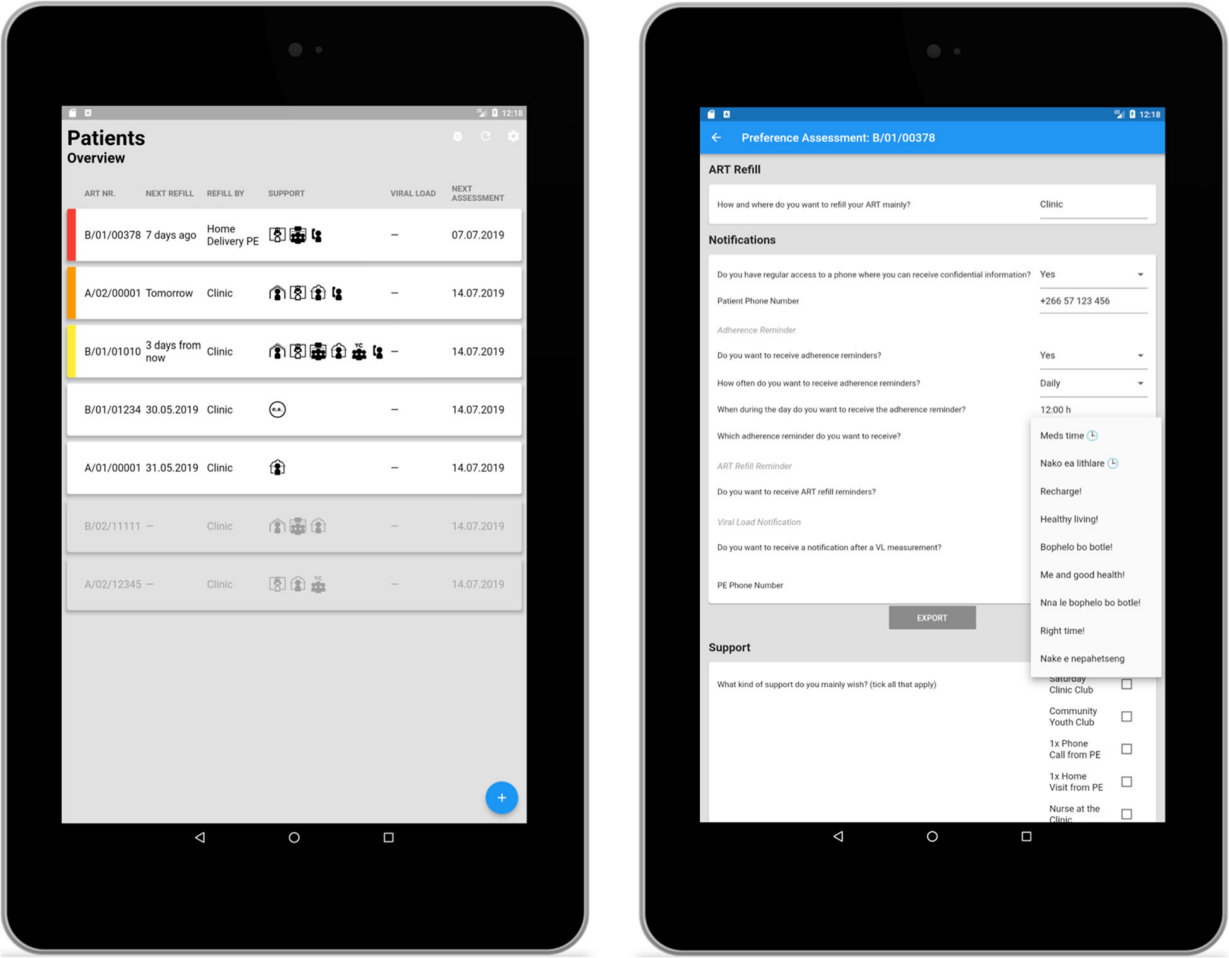
PEBRApp

The participants in the intervention clusters are offered the PEBRA model. In the PEBRA model, the ART services are coordinated by the PE as much as feasible and depending on the participants’ preferences regarding medication pick-up, SMS notifications and support options. Details are outlined in Fig. 1. First, the PE explains all options to the participant who then chooses according to his/her preferences. Thereafter, the PE systematically assesses the feasibility of the option chosen as not all options are available to everyone all the time, e.g. no nearby Village Health Worker (VHW) available who could dispense ART, or no community youth club established in the participants’ community, or home-delivery by the PE not being feasible. Finally, the compromise between preference and feasibility is delivered. The preferences are assessed at enrolment and thereafter follow a strict schedule: every month for participants with unsuppressed VL (> 1000 copies/mL) and every 3 months for participants with suppressed VL. The PE delivers the PEBRA model using a tablet-based application, called PEBRApp (Fig. 2). The PEBRApp helps the PE to assess the preferences, to deliver them in a feasible manner, to keep track of the ART refill and next assessment dates, and to ensure regular contact between the PE and the participant. The PEBRApp is protected by a password and does not entail confidential patient information, i.e. names. The chosen SMS notifications are sent automatically through an external platform, and always include a call-back option to the PE’s number.

The PEBRA model and PEBRApp were designed in collaboration with peer-educators, AYPLHIV, youth advocates, clinical staff and application developers during several workshops supported and coordinated by two local non-profit organizations (SolidarMed & Sentebale) as well as the Ministry of Health of Lesotho. The model incorporates the existing support structures at the health facilities. Sentebale is running a long-standing PE program in collaboration with the Ministry of Health. The study PEs are recruited from this existing PE program. They are trained young people living in a community of their respective health facility catchment area. All PEs involved in PEBRA trial receive an additional training on the PEBRApp and use of the tablet, referral and documentation system, obtaining informed consent and other study-specific procedures. Besides their usual close supervision by the health facility staff and Sentebale they are supervised by the study staff with regular onsite monitoring visits.

### Outcomes

The primary endpoint is viral suppression at 12 months, defined as the proportion of participants in care with a documented VL < 20 copies/mL 12 months (range: 9–15 months) after enrolment out of all participants enrolled, including participants who transferred out to any other health facility with documented proof of a VL laboratory report in the endpoint window. The secondary endpoints are defined in Table 1. Furthermore, qualitative research to explore the acceptability of the PEBRA model and a cost-effectiveness analysis to estimate the impact of PEBRA model on health outcomes and costs will be conducted.

**Table 1:** Secondary endpoints of PEBRA trial

Secondary endpoints	***Definition***	***Time point following enrolment***	***Remarks***
Engagement in care	Proportion of all participants engaged in care	6 months (range 5–8) and 12 months (range 9–15)	*Definition of “in care”: at least one ART visit in the defined window* I. *Including participants who transferred out to any other health facility with known outcome (documented proof of follow-up visit or laboratory test)*
All cause mortality	Proportion of all participants who died	6 months (range 5–8) and 12 months (range 9–15)	*Verbal autopsy to capture cause of death whenever possible. No death certificate or autopsy report required.*
Lost-to-follow-up (LTFU)	Proportion of all participants LTFU	6 months (range 5–8) and 12 months (range 9–15)	*We define participants lost to follow-up if they or their treatment buddies were more than 2 months late for a scheduled consultation or medication pick-up and no information was found about the participant*
Transfer out (TO)	Proportion of all participants who transferred out to any other health facility (than the initially attached one) with known outcome	6 months (range 5–8) and 12 months (range 9–15)	*Definition of “known outcome”: Documented proof of follow-up visit or laboratory test of the new health facility*
Viral suppression < 1000 copies/ml	Proportion of all participants with viral suppression (< 1000 copies/mL)	12 months (range: 9–15)	*Some of the remote health facilities in the study districts face regular challenges in sending the blood to the government hospital. To ensure sufficient VL measurements among study participants, these health facilities will be equipped with dried-blood-spot (DBS) as a backup for VL measurement. According to the WHO the recommended threshold for treatment failure using DBS is 1000 copies/mL*
Adherence	Assessed by 3 different setting- and age-validated ART self-reported adherence questions: 1. “When was the last time you missed any medications?” [i) past week, ii) 1–2 weeks ago, iii) 3–4 weeks ago, iv) never]: Dichotomous outcome missed doses vs. no missed doses in the past month 2. “ART missed at two or more consecutive days within last month?”: Dichotomous outcome 3. “How would you rate your adherence over the last month” [i) very poor, ii) poor, iii) fair, iv) good, v) very good, vi) excellent]: Dichotomous outcome adherent vs non-adherent (anything less than ‘excellent’)	3 months (range 2.5–3.5), 6 months (range 5–8) and 12 months (range 9–15)	
Quality of life	Assessed by SF-12 (Likert-Scale)	6 months (range 5–8) and 12 months (range 9–15)	
Satisfaction of care	Assessed by a self-reported patient service satisfaction questionnaire partly based on a setting-validated quality of care questionnaire (Likert-Scale)	6 months (range 5–8) and 12 months (range 9–15)	*Assessed by an external data collector, not the PE*

### Data collection and management

**Fig. 3:**
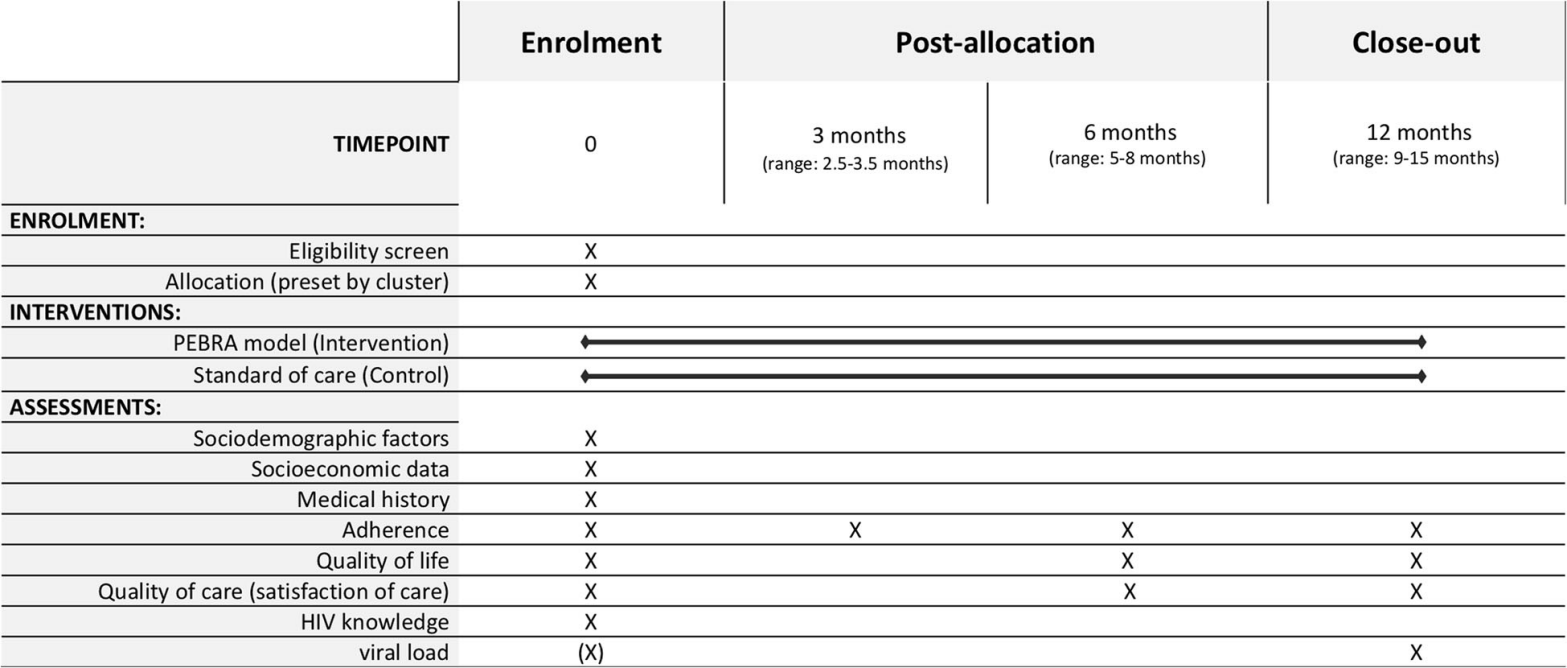
PEBRA trial SPIRIT diagram

At enrolment, the PE administers a questionnaire integrated in the PEBRApp. Questionnaire domains include sociodemographic and socioeconomic data, medical history, HIV/AIDS knowledge, adherence, quality of life and satisfaction of care. Follow-up data is collected using PEBRApp according to the schedule outlined in Fig. 3. Baseline VL is defined as the last VL within the previous 12 months. If no VL within previous 12 months available, then the participant is sent to the nurse for routine VL measurement at enrolment.

The PEBRApp is password-protected and all data is regularly backed up into at a password-protected database. Similarly, relevant data for the SMS intervention is uploaded to a separate encrypted and password-protected online database that offers the possibility to send out SMS automatically and is connected to the district laboratory database containing the VL results. SMS are dispatched using the trusted third-party provider Twilio, certified with the privacy shield framework. Access to all data collection tools and databases are strictly limited and regulated through personal user profiles. The PEBRA trial represents implementation research without any experimental medication and only well-established ART in Lesotho according to the National guidelines. Thus, we do not expect serious adverse effects (SAE) on patients’ health from this intervention. However, for the purpose of this trial, we will capture the following SAEs: a) life-threatening event, b) hospitalization, c) persistent or significant disability or incapacity, d) congenital anomaly / birth defect, e) death. It is not planned to establish a data safety and monitoring board.

### Sample size and analysis

Based on cohort and program data from the study districts we expect on average 15 AYPLHIV per health facility with an overall viral suppression rate (< 20 copies/ml) of 70%. An overall target sample size of 300 AYPLHIV in 20 clusters would provide 90% power to detect a 20% increase of viral suppression in the intervention compared to the control group, assuming a type 1 error of 0.05. An intra-cluster correlation coefficient (ICC) of 0.05 (design effect of 1.7) was estimated based on similar studies [28, 29].

Analyses are performed following the CONSORT guidelines for cluster-randomized trials and an intention-to-treat principle including all participants as randomized per cluster randomization. Clusters are the unit of randomization, but individuals are the unit of analysis. The primary analysis uses random effects logistic regression models including cluster as a random effect and arm allocation as a fixed effect to assess the difference between viral suppression rate in the intervention versus control arm. The model will be further adjusted for the stratification factor, and relevant baseline factors that may be randomly unbalanced between intervention and control clusters. All results are presented with odds ratios and their respective 95% confidence intervals. Categorical variables will be described with absolute and relative frequencies and continuous variables as medians and interquartile ranges. In pre-specified subgroup analyses, the effect of gender, age groups, education, marital status, ART regimen and HIV/AIDS knowledge on key study outcomes will be assessed by including interaction terms in the model. If an interaction term is found to be significant, effect estimates will be summarized by subgroup. As the study is not powered for these pre-planned subgroup analyses, these results will be considered exploratory. Where substantial data are missing in important covariates, multiple imputation will be utilized and the results compared to models ignoring missing data. A sensitivity analysis will be performed to assess the effect of baseline viral load on the primary endpoint, only including participants for whom this data is available. All analyses are done using Stata (version 14, Stata Corporation, Austin, Texas, USA), using two-sided *p*-values and a significance level of 0.05.

## Discussion

Effective and differentiated strategies are needed to improve the HIV care cascade among adolescents and young adults, especially in rural settings in SSA. Although many countries in SSA have some sort of PE program in place, there is lack of published high-quality evidence for adolescent-specific DSD models involving peers [20].

The Zvandiri DSD model in rural Zimbabwe introduced community adolescent treatment supporters to offer facility-based as well as community-based support for AYPLHIV, including piloting ART delivery through peers. In a small randomized clinical trial the model improved self-reported adherence to ART, engagement in care, and psychosocial well-being [24]. This effect could no longer be observed at 2 years follow-up in a subsequent larger cluster-randomized trial, however, a 40% reduction in virologic failure was documented among participants in the model [25]. The questions remain, though, to what extent DSD models can be individualized and peers can be involved in clinical care. In recent years there has been an increasing programmatic shift towards DSD models, however they usually have narrow inclusion criteria and limited options to choose from. The PEBRA model tries to incorporate the existing structures and support options provided by different stakeholders, differentiates the DSD by personal preferences and puts the PE at the heart of the model.

This trial has several limitations. First, as in most operational research studies, we will have only limited control over what happens in our standard care clusters. Second, due to the nature of this pragmatic implementation trial, it is not possible to blind participants or staff to the intervention. Third, it was not feasible to perform the randomization after recruitment of the participants. That means, there is a possibility of recruitment bias. To mitigate this risk, two different consent forms for control versus intervention arm concealing the exact intervention of the other arm were used, thus the participants were aware of being in a study, but not of being in a trial.

The PEBRA trial will provide evidence on the feasibility and effectiveness of an inclusive and holistic DSD model for AYPLHIV that is more preference-based and coordinated by a PE. If proven to be effective, the PEBRA model and PEBRApp have the potential to be scaled up in similar settings.

## Trial status

The trial was launched on November 4, 2019. The study is ongoing, and we expect to reach the required minimum target sample size within 5 months. The questionnaires, PEBRApp and the PEBRA model were pretested in a pilot trial from August – October 2019. The pilot trial assessed the acceptance of the PEBRA model and its delivery by the PE, the technical functionalities of PEBRApp and the questionnaires.

## Data Availability

The datasets used and analysed during the trial are available from the corresponding author on reasonable request.

## Abbreviations

ART: Antiretroviral Therapy
AYPLHIV: Adolescents & Young People Living with HIV
CAC: Community Adherence Clubs
CI: Confidence Interval
CYC: Community Youth Club
DHMT: District Health Management Team
DSD: Differentiated Service Selivery
HIV: Human Immunodeficiency Virus
ICC: Intra-cluster correlation coefficient
LTFU: Loss-To-Follow-Up
OR: Odds Ratio
PE: Peer-Educator
PEBRA: Peer-Educator-Based Refill of ART
SAE: Serious Adverse Event
SSA: Sub-Saharan Africa
VHW: Village Health Worker
VL: Viral Load (Plasma HIV-1 RNA)
WHO: World Health Organization

## References

[1] UNAIDS data 2019 [Internet]. Available from: https://www.unaids.org/en/resources/documents/2019/2019-UNAIDS-data [cited 2019 25 Nov].

[2] UNICEF HIV and AIDS data 2017 [Internet]. Available from: https://www.unicef.org/publicpartnerships/files/Annual_Results_Report_2017_HIV_And_AIDS.pdf. [cited 2020 20 Mar].

[3] Davies M-A, Pinto J (2015). Targeting 90–90–90 – don’t leave children and adolescents behind. J Int AIDS Soc.

[4] Slogrove AL, Mahy M, Armstrong A, Davies M-A. Living and dying to be counted: What we know about the epidemiology of the global adolescent HIV epidemic. J Int AIDS Soc. 2017;20 Available from: https://onlinelibrary.wiley.com/doi/abs/10.7448/IAS.20.4.21520 [cited 2019 25 Feb]. 10.7448/IAS.20.4.21520 PMC5719718 28530036

[5] Bekker L-G, Siberry GK, Hirnschall G (2018). Ensuring Children and Adolescents Are Not Left Behind. JAIDS J Acquir Immune Defic Syndr.

[6] UNAIDS and UNICEF data 2018 [Internet]. Available from: https://www.childrenandaids.org/sites/default/files/2018-07/ALL-IN-in-Eastern-and-Southern-Africa-WEB_2018.pdf. [cited 2020 20 Mar].

[7] Auld AF, Agolory SG, Shiraishi RW, Wabwire-Mangen F, Kwesigabo G, Mulenga M (2014). Antiretroviral therapy enrollment characteristics and outcomes among HIV-infected adolescents and young adults compared with older adults--seven African countries, 2004-2013. MMWR Morb Mortal Wkly Rep.

[8] Plummer ML, Baltag V, Strong K, Dick B, Ross DA, World Health Organization, et al. Global Accelerated Action for the Health of Adolescents (AA-HA!): guidance to support country implementation [Internet]. 2017 [cited 2019 Feb 25]. Available from: http://apps.who.int/iris/bitstream/10665/255415/1/9789241512343-eng.pdf.

[9] Hudelson C, Cluver L (2015). Factors associated with adherence to antiretroviral therapy among adolescents living with HIV/AIDS in low- and middle-income countries: a systematic review. AIDS Care.

[10] Govindasamy D, Ford N, Kranzer K (2012). Risk factors, barriers and facilitators for linkage to antiretroviral therapy care: a systematic review. AIDS.

[11] Hall BJ, Sou K-L, Beanland R, Lacky M, Tso LS, Ma Q (2017). Barriers and facilitators to interventions improving retention in HIV care: a qualitative evidence meta-synthesis. AIDS Behav.

[12] McNairy ML, El-Sadr WM (2012). The HIV care continuum: no partial credit given. AIDS Lond Engl.

[13] Joint United Nations Programme on HIV/AIDS. Miles to go: Closing gaps, breaking barriers, righting injustices. Geneva, Switzerland; UNAIDS; 2018. Available from: http://www.unaids.org/sites/default/files/media_asset/miles-to-go_en.pdf [cited 2019 25 Feb].

[14] Kanters S, Park JJH, Chan K, Socias ME, Ford N, Forrest JI, et al. Interventions to improve adherence to antiretroviral therapy: a systematic review and network meta-analysis. Lancet HIV. 4(1):e31–40 Available from: http://dx.doi.org/10.1016/S2352-3018(16)30206-5 [cited 2017 13 Sep]. 10.1016/S2352-3018(16)30206-5 27863996

[15] International AIDS Society. Differentiated Care for HIV: A Decision Framework for Antiretroviral Therapy. Durban, South Africa: IAS; 2016. Available from: http://www.differentiatedcare.org/Portals/0/adam/Content/yS6M-GKB5EWs_uTBHk1C1Q/File/Decision%20Framework.pdf [cited 2019 25 Feb].

[16] WHO/IAS data 2018. Providing differentiated delivery to children and adolescents [Internet]. Available from: https://www.who.int/hiv/pub/paediatric/diff-delivery-children-hiv/en/. [cited 2020 20 Mar].

[17] Grimsrud A, Bygrave H, Doherty M, Ehrenkranz P, Ellman T, Ferris R (2016). Reimagining HIV service delivery: the role of differentiated care from prevention to suppression. J Int AIDS Soc.

[18] PATA Technical Brief about Differentiated Service Delivery for Adolescents and Young Adults Living with HIV in South Africa 2019 [Internet]. Available from: http://teampata.org/wp-content/uploads/2019/02/DSD_Policy-Brief_2019.pdf. [cited 2020 20 Mar].

[19] A Decision Framework for differentiated antiretroviral therapy delivery for children, adolescents and pregnant and breastfeeding women. International AIDS Society 2017 [Internet]. Available from: http://www.differentiatedcare.org/Portals/0/adam/Content/9ErIJtsSfUmj_Ska6BoN0Q/File/Decision%20Framework%20for%20children%20adolescents%20and%20pregnant%20and%20breastfeeding%20women.pdf. [cited 2020 20 Mar].

[20] WHO data 2019. Adolescent-friendly health services for adolescents living with HIV: from theory to practice [Internet]. Available from: https://www.who.int/publications-detail/adolescentfriendly-health-services-for-adolescents-living-with-hiv. [cited 2020 20 Mar].

[21] MacKenzie RK, van Lettow M, Gondwe C, et al. Greater retention in care among adolescents on antiretroviral treatment accessing ‘Teen Club’ an adolescent-centred differentiated care model compared with standard of care: a nested case-control study at a tertiary referral hospital in Malawi. J Int AIDS Soc. 2017. p. 20. 10.1002/jia2.25028. 10.1002/jia2.25028 PMC5810310 29178197

[22] Funck-Brentano I, Dalban C, Veber F, Quartier P, Hefez S, Costagliola D (2005). Evaluation of a peer support group therapy for HIV-infected adolescents. AIDS Lond Engl..

[23] Tsondai PR, Wilkinson LS, Grimsrud A, Mdlalo PT, Ullauri A, Boulle A. High rates of retention and viral suppression in the scale-up of antiretroviral therapy adherence clubs in Cape Town, South Africa. J Int AIDS Soc. 2017;20:21649. 10.7448/IAS.20.5.21649 PMC5577696 28770595

[24] Willis N, Milanzi A, Mawodzeke M, Dziwa C, Armstrong A, Yekeye I (2019). Effectiveness of community adolescent treatment supporters (CATS) interventions in improving linkage and retention in care, adherence to ART and psychosocial well-being: a randomised trial among adolescents living with HIV in rural Zimbabwe. BMC Public Health.

[25] Zvandiri Trial Policy Brief 2019 [Internet]. Available from: http://www.differentiatedcare.org/Portals/0/adam/Content/4QG5cuSbCkmSdl9p11ywUQ/File/Zvandiri%20Trial%20Policy%20Brief%207%20August%202019.pdf. [cited 2020 20 Mar].

[26] UNAIDS. Prevention Gap Report. 2016 [Internet]. Available from: http://www.unaids.org/sites/default/files/media_asset/UNAIDS_Gap_report_en.pdf. [cited 2016 10 Apr].

[27] LePHIA report 2016-2017 [Internet]. Available from: https://phia.icap.columbia.edu/wp-content/uploads/2018/02/Lesotho-Summary-Sheet_A4.2.7.18.HR_.pdf. [cited 2020 20 Mar].

[28] Elul B, Lamb MR, Lahuerta M, Abacassamo F, Ahoua L, Kujawski SA (2017). A combination intervention strategy to improve linkage to and retention in HIV care following diagnosis in Mozambique: A cluster-randomized study. PLOS Med.

[29] McNairy ML, Lamb MR, Gachuhi AB, Nuwagaba-Biribonwoha H, Burke S, Mazibuko S (2017). Effectiveness of a combination strategy for linkage and retention in adult HIV care in Swaziland: The Link4Health cluster randomized trial. PLOS Med.

